# Improvement of drug‐induced gingival overgrowth and cerebrovascular related dementia after dental treatments

**DOI:** 10.1002/ccr3.8093

**Published:** 2023-10-23

**Authors:** Naomi Tanoue, Hanako Kawasaki, Kensuke Kiriishi, Takao Ayuse

**Affiliations:** ^1^ Nagasaki University Graduate School of Biomedical Sciences Division of Pediatric Dentistry Nagasaki Japan; ^2^ Department of Special Care Dentistry Nagasaki University Hospital Nagasaki Japan; ^3^ Division of Clinical Physiology Nagasaki University Graduate School of Biomedical Sciences Nagasaki Japan

**Keywords:** amlodipine, dental occlusion, gingival overgrowth, mental status and dementia tests, phenytoin

## Abstract

Drug‐induced gingival overgrowth can occur as a side effect of specific drugs and lead to poor oral function. Appropriate dental management of the overgrowth may improve oral function and improve cognitive deficits after cerebrovascular accidents.

## Graphical abstract

The side effects of the drug caused gingival overgrowth and destruction of oral function.

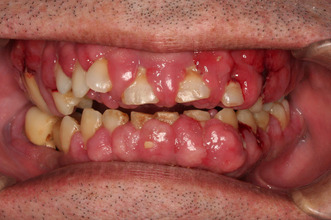

## 1 INTRODUCTION

Drug‐induced gingival overgrowth (DIGO) is well known as a side effect associated principally with three types of drugs: anticonvulsants (e.g., phenytoin), immunosuppressants (e.g., cyclosporine A), and various calcium channel blockers (e.g., nifedipine, Amlodipine, diltiazem).^1^, ^2^, ^3^ Although the mechanisms of action of each can be expected to differ, the clinical and microscopic appearance of DIGO due to any of these drugs is quite similar.

DIGO tends to be more severe in areas where plaque accumulates. Daily plaque control is therefore important in managing gingival enlargement, in addition to professional periodontal treatment. However, in some cases of DIGO, plaque control cannot always be achieved. Daily periodontal care can be quite difficult for individuals with dementia who may not understand the importance of such care. DIGO is prone to relapse in such individuals, and if a gingivectomy and scaling are necessary, one option is to perform these procedures with the patient under intravenous sedation or general anesthesia.

Medical management that includes reducing a medication's dose or changing to another drug is another method to bring about the regression of the lesion. Dentists can consult a patient's primary care physician and ask for a reduction in the patient's medication dosage or a change of a gingival enlargement‐inducing medication. If such medical management is possible, most of the patients treated in this manner will observe an alteration in the soft tissues. Adequate medical management and dental treatment may improve not only dental diseases but also systemic diseases to an extent that is better than expected. We report herein the case of our patient with vascular dementia, which improved with the dental treatment of his DIGO.

## 2 CASE PRESENTATION

A 58‐year‐old Japanese male presented to the Department of Special Care Dentistry at Nagasaki University Hospital because his gingival hyperplasia/bleeding and bad breath had been reported by the staff of the facility for the disabled where he had been staying. His medical history included hypertension, dementia, and post‐stroke epilepsy due to a cerebral vascular accident (CVA) that had occurred 11 years earlier. His drug regimen was as follows: Phenytoin (320 mg/day; i.e., 3.2 g/day of Aleviatin®), Amlodipine (2.5 mg/day), amantadine hydrochloride (100 mg/day), sodium azulene sulfonate (2 g/day), lansoprazole (15 mg/day), and heavy magnesium oxide (1 g/day). There was no family history of gingival overgrowth.

As an aftereffect of the patient's CVA, his disturbance of motility (left hemiplegia) was significant. His score on the Hasegawa's Dementia Scale‐Revised (HDS‐R) was 12/30; reduced cognitive function is usually defined as a score of 20/30 or lower.^4^ He was uncooperative with all aspects of dental care and treatment and often became violent despite his disorder. He refused any oral examination.

**FIGURE 1:**
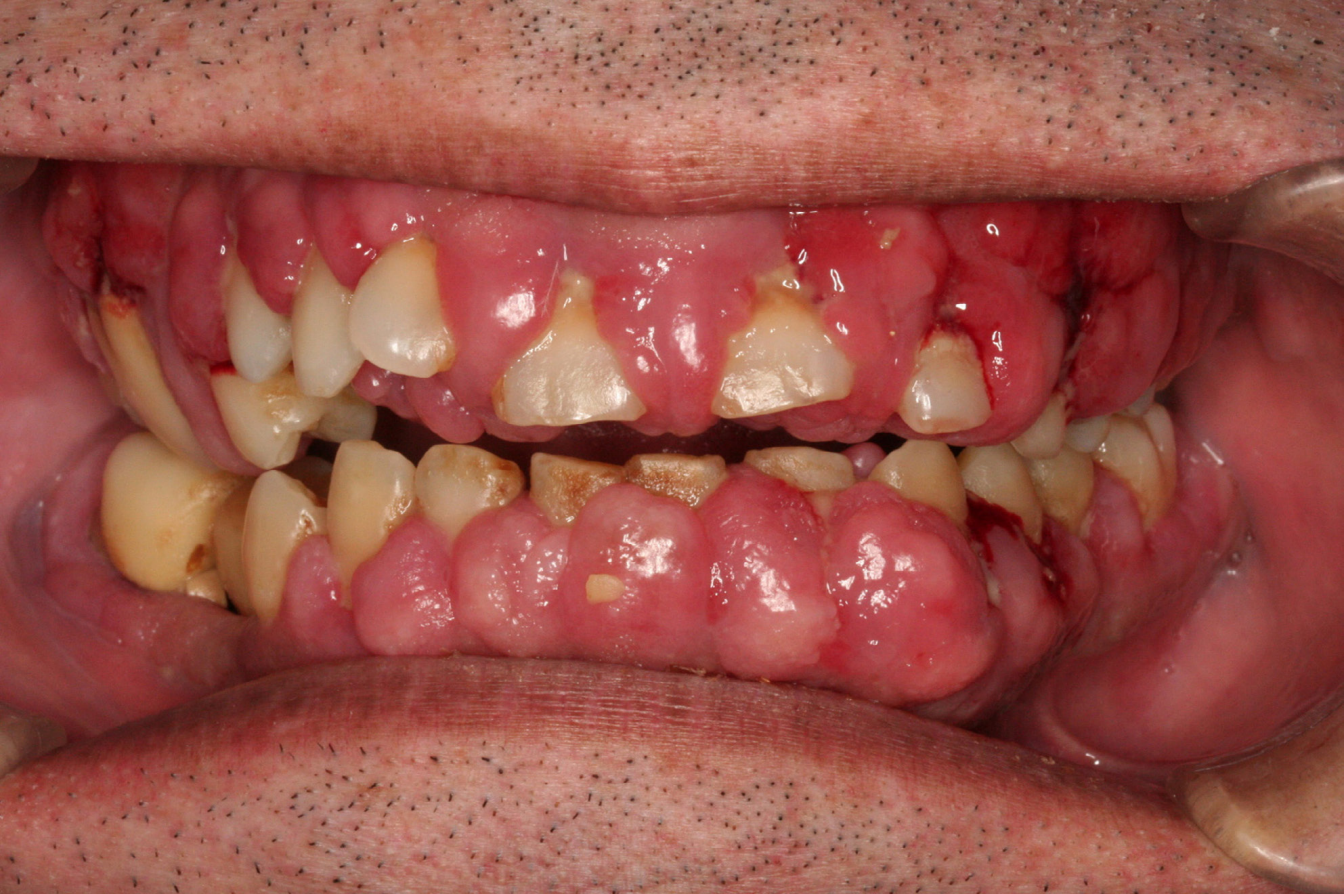
Frontal view at the second visit (the first examination using intravenous anesthesia). Twothirds of the tooth crown length was covered by the gingiva.

An intraoral examination using intravenous anesthesia revealed that approximately twothirds of his tooth crown length was covered by the gingiva (Figure 1). Plaque was present on all surfaces, and the inflammation of the gingiva was severe. The periodontal pocket depth was >8 mm, and gingival bleeding was observed for all teeth. Only the molars were occluded, but their mobilities were all Class III. The lesion was diagnosed as DIGO based on the patient's his drug history and the intra‐oral examination.

**FIGURE 2:**
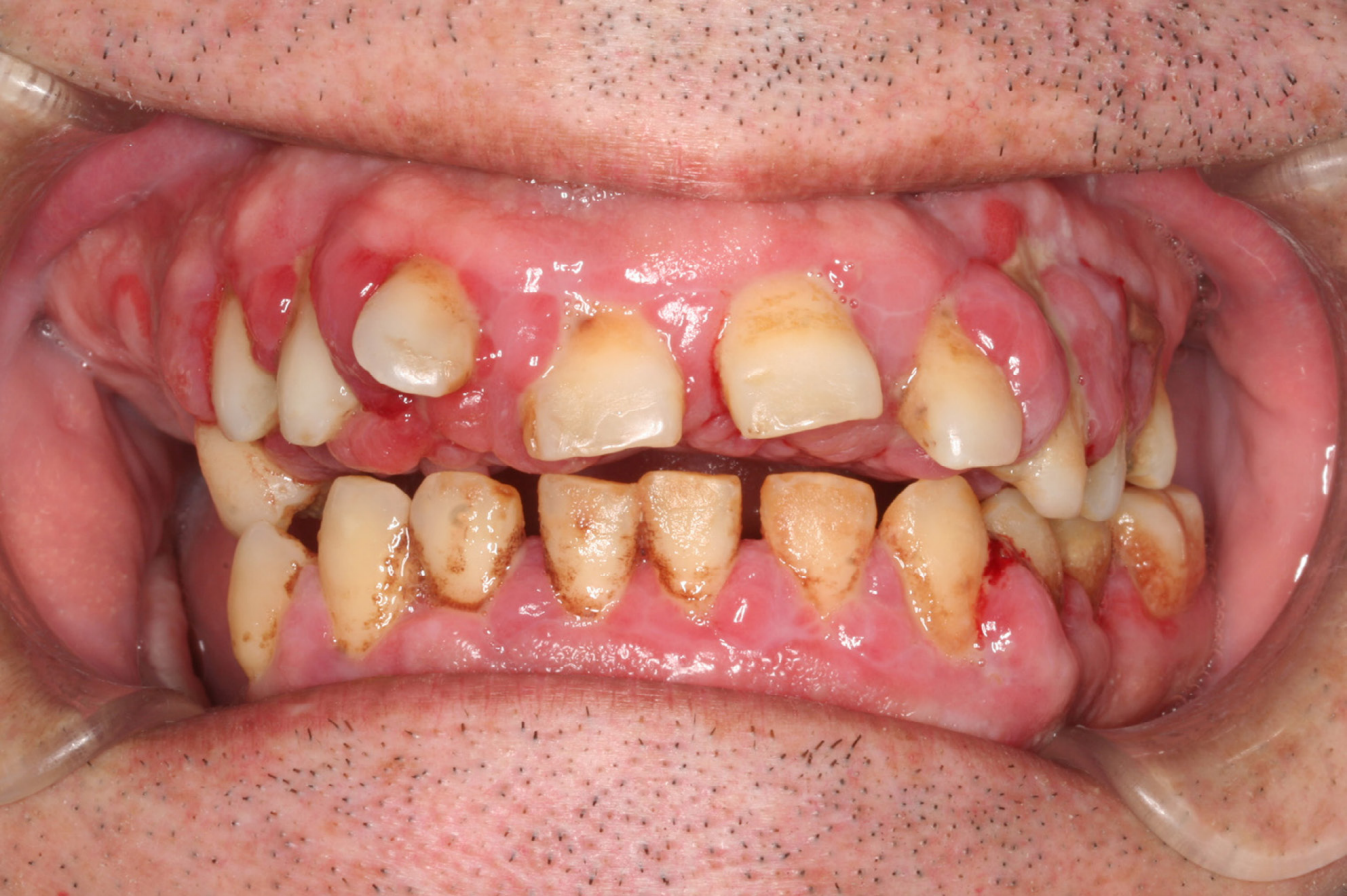
Frontal view at the fourth visit (the fifth examination using intravenous anesthesia). The gingiva was surgically treated, but strong inflammatory findings were noted.

At the first dental examination using intravenous anesthesia (second visit), seven floating molars [17, 16, 26, 27, 37, 46, and 47] were extracted. At the third and fourth visits, a gingivectomy using an electric scalpel and scaling were performed with the patient under intravenous anesthesia. The gingivitis then improved slightly, but swelling and bleeding continued (Figure 2). The patient's HDS‐R score was 11–12 at that time, as at the first and second visits.

At the patient's fifth visit, in addition to gingivectomy and scaling, occlusal adjustments of premolars were performed to increase the contact area at the central occlusal position. We then asked the patient's primary care physician to manage the two types of drug (amlodipine and phenytoin) that we suspected to be the cause of his DIGO. The physician changed the patient's amlodipine (2.5 mg/day) to olmesartan (20 mg/day) and reduced his phenytoin dosage (320–100 mg/day).

**FIGURE 3:**
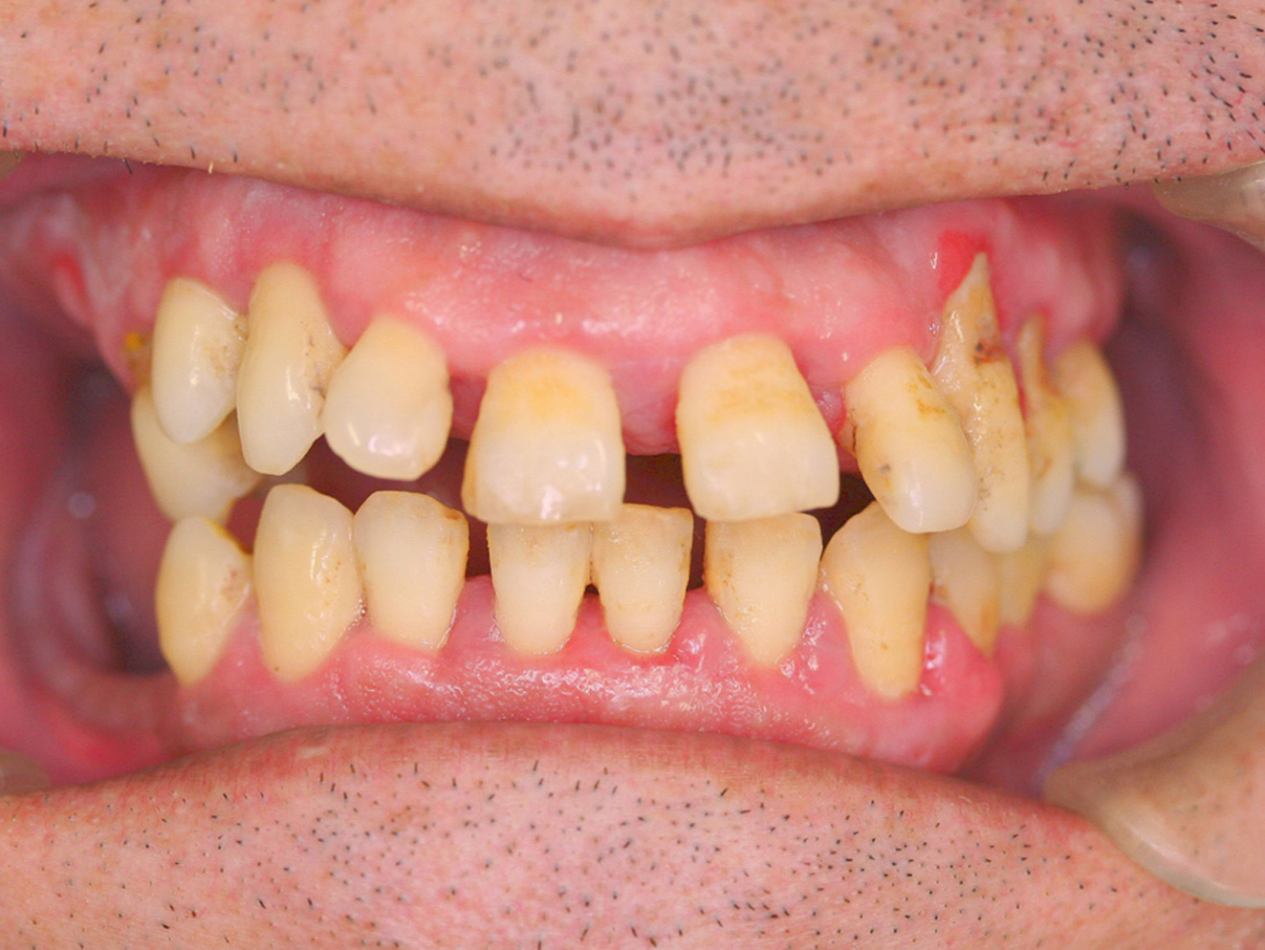
Frontal view at the fifth visit (the sixth examination using intravenous anesthesia) after occlusal adjustment and drug management. The gingiva had been significantly improved and the inflammatory findings have almost disappeared. Occlusion had also been improved.

**FIGURE 4:**
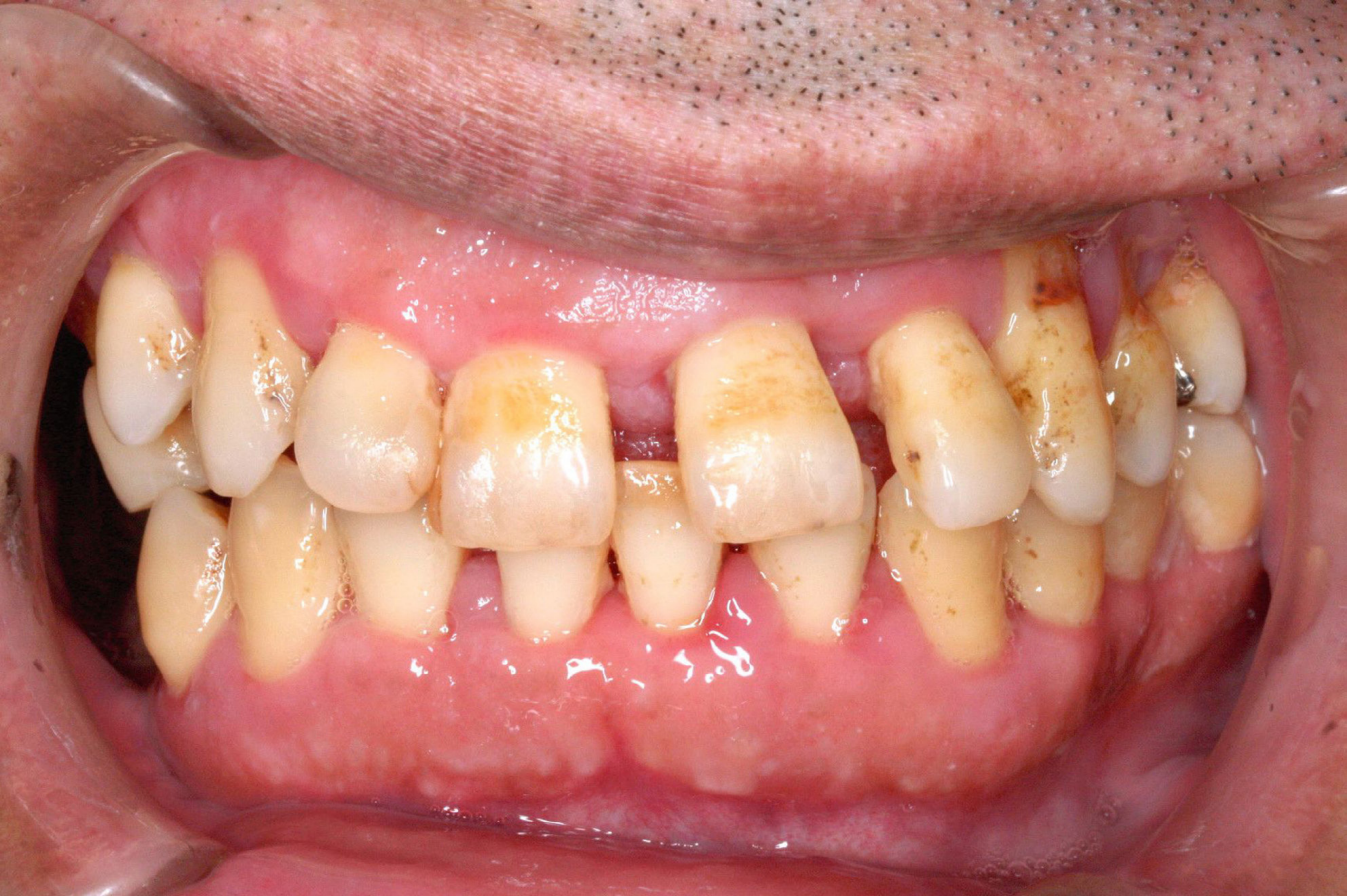
Frontal view at the sixth visit (follow‐up under consciousness). The inflammatory findings have disappeared.

At the sixth visit the next month, only general periodontal treatments and occlusal adjustment were performed with the patient under intravenous anesthesia, since the gingival swelling was significantly alleviated (Figure 3). At the seventh visit, when the patient was recalled 4 months later for a follow‐up without intravenous anesthesia, he showed complete recovery of the DIGO (Figure 4). He had also become able to cooperate with minimally invasive dental treatments, and his violent behavior had decreased. The patient's HDS‐R score at that time had risen significantly to 23/30, and he could speak a little.

## 3 DISCUSSION

For the treatment of DIGO, medical management should be carried out carefully along with the general dental treatments including gingival surgery, professional debridement with scaling and root planning, and tooth brushing instruction. Fortunately, it was possible to reduce and switch the gingival enlargement‐inducing drugs that had been used to treat our patient with dementia, and doing so dramatically improved his DIGO.

Surprisingly, the patient's HDS‐R score was also improved after the dental treatments. His CVA had occurred 11 years earlier, and his cerebrovascular related dementia had been diagnosed at the dental visits. Although the physiological mechanism underlying the improvement of our patient's HDS‐R score cannot be proposed based on solely the present case, we speculate that there were three factors involved in the improvement of his dementia following dental treatment. One factor is the recovery of the body's systemic condition by the removal of oral infection sources by tooth extraction and periodontal treatment. Poor oral health is known to influence the initiation and/or progression of neurodegenerative diseases.^5^

As a second factor, the cerebral blood flow might be increased by the improvement of occlusion. Occlusal stimulation has been reported to increase cerebral blood flow and improve dementia.^6^ In addition, a clear relationship between the number of pairs of antagonist teeth that are in contact while an individual's mouth is closed and his or her cognitive impairment level was reported.^7^

The third factor is the possibility that a change in a medication (in our patient's case, from amlodipine to olmesartan) directly affects the improvement of dementia. The mechanism of action is likely to differ by the type of medication, even in the same class of antihypertensive agents. However, it seems unlikely that the olmesartan in our patient's case was solely responsible for the improvement of his dementia. The reduction of phenytoin's sedative effect (by reducing the daily dose from 320 to 100 mg) may also have contributed to the dementia improvement.

**FIGURE 5:**
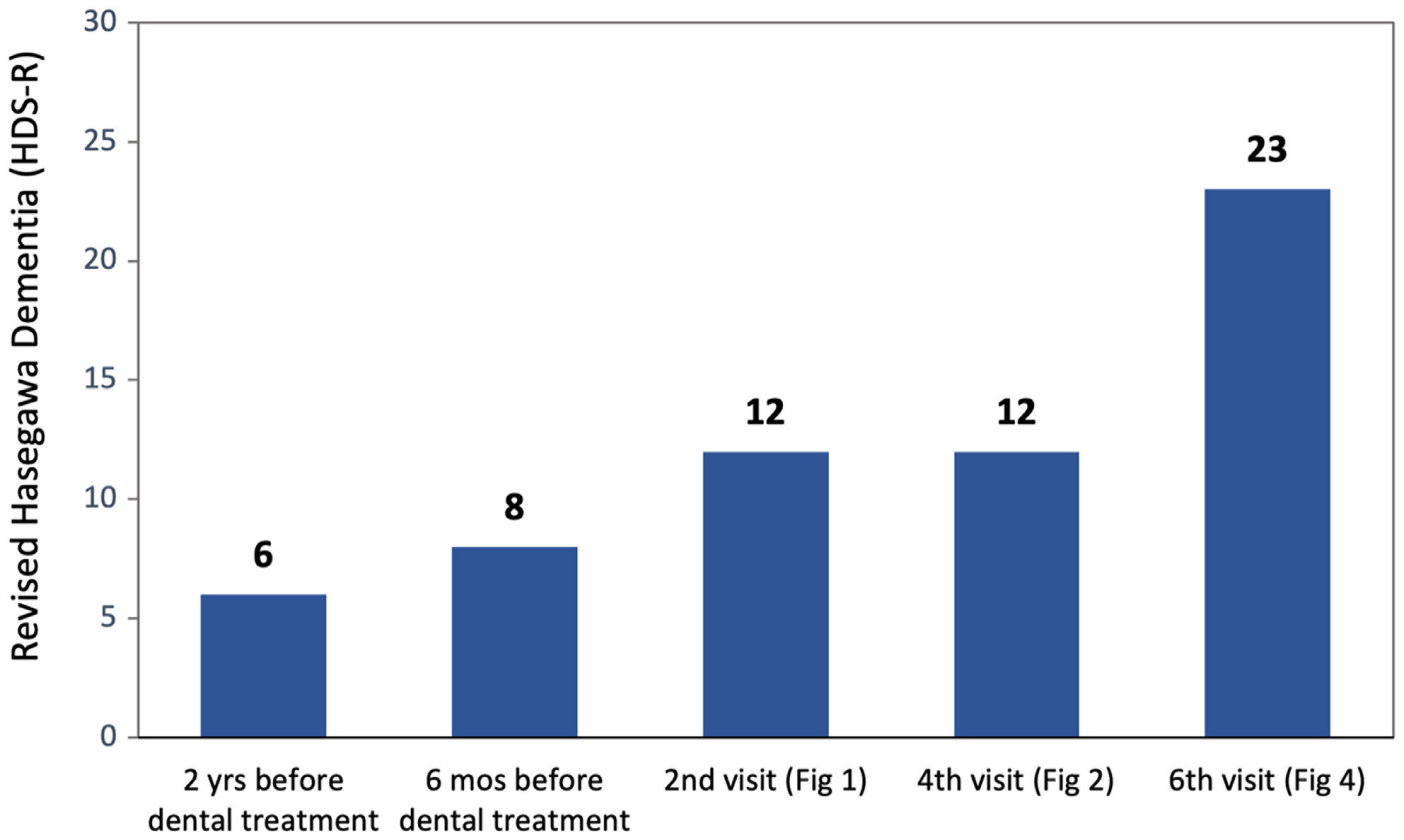
Transition of HDS‐R. It can be seen that score values increased immediately after occlusion and drug managements.

The present case does not confirm that the medical and dental treatments were responsible for the patient's dementia. His HDS‐R score was already showing a tendency to increase before his dental treatment (Figure 5). Nevertheless, the patient's DIGO, his HDS‐R score, and his ability to cooperate with medical/dental procedures definitely improved simultaneously, within a short period. His case highlights the importance of dental treatment for improving and maintaining systemic conditions.

## AUTHOR CONTRIBUTIONS

**Naomi Tanoue:** Conceptualization; methodology; validation; writing – original draft. **Hanako Kawasaki:** Visualization. **Kensuke Kiriishi:** Supervision. **Takao Ayuse:** Supervision; writing – review and editing.

## FUNDING INFORMATION

The authors received no financial support for the research.

## CONFLICT OF INTEREST STATEMENT

The authors declare no conflicts of interest associated with this manuscript.

## ETHICS STATEMENT

This case report was approved by the Nagasaki University Hospital Clinical Research Ethics Committee in April, 2023 (Approval Number 23041727).

## CONSENT

Oral and written informed consent was obtained from the patient to publish this report in accordance with the journal's patient consent policy.

## Data Availability

The data that support the findings of this study are available from the corresponding author, [NT], upon reasonable request.

## References

[1] Al‐Hamilly NS , Radwan LR , Abdul‐Rahman M , Mourad MI , Grawish ME . Biological roles of KGF, CTGF and TGF‐β in cyclosporine‐A‐ and phenytoin‐induced gingival overgrowth: a comparative experimental animal study. Arch Oral Biol. 2016;66:38‐43. doi:10.1016/j.archoralbio.2016.02.006 26894526

[2] Guollo A , Vivas AP , Lopes RN , Porta G , Alves FA . Amlodipine‐induced gingival overgrowth in a child after liver transplant. Autops Case Rep. 2016;6(3):47‐51. doi:10.4322/acr.2016.041 PMC5087984 27818959

[3] Anand AJ , Gopalakrishnan S , Karthikeyan R , Mishra D , Mohapatra S . Immunohistochemical analysis of the role connective tissue growth factor in drug‐induced gingival overgrowth in response to phenytoin, cyclosporine, and nifedipine. J Int Soc Prev Commun Dent. 2018;8(1):12‐20. doi:10.4103/jispcd.JISPCD_403_17 PMC5853037 29629324

[4] Kim KW , Lee DY , Jhoo JH , et al. Diagnostic accuracy of mini‐mental status examination and revised Hasegawa dementia scale for Alzheimer's disease. Dement Geriatr Cogn Disord. 2005;19(5–6):324‐330. doi:10.1159/000084558 15785033

[5] Scannapieco FA , Cantos A . Oral inflammation and infection, and chronic medical diseases: implications for the elderly. Periodontol 2000 2016;72(1):doi:10.1111/prd.12129, 153, 175. 27501498

[6] Ono Y , Yamamoto T , Kubo KY , Onozuka M . Occlusion and brain function: mastication as a prevention of cognitive dysfunction. J Oral Rehabil. 2010;37(8):624‐640. doi:10.1111/j.1365-2842.2010.02079.x 20236235

[7] Cardoso MG , Diniz‐Freitas M , Vázquez P , Cerqueiro S , Diz P , Limeres J . Relationship between functional masticatory units and cognitive impairment in elderly persons. J Oral Rehabil. 2019;46(5):417‐423. doi:10.1111/joor.12763 30614023

